# Impacts of feeding milk replacer containing 20% or 24% protein and fat on growth and feed efficiency of beef × dairy crossbred calves

**DOI:** 10.3168/jdsc.2024-0726

**Published:** 2025-05-11

**Authors:** Jason D. Stypinski, Andrew L. Plumski, Dave M. Ziegler, William. P. Hansen, Mark F. Scott, Isaac J. Salfer

**Affiliations:** 1 Department of Animal Science, University of Minnesota, Saint Paul, MN 55108; 2 Southern Research and Outreach Center, University of Minnesota, Waseca, MN 56093; 3 Actus Nutrition, Eden Prairie, MN 55344

## Abstract

It is becoming more prominent for dairy producers to breed their lower genetic merit cows and heifers to beef sires to increase the value of calves when they are sold to beef markets. However, data are currently lacking on optimal feeding strategies for rearing these crossbred calves until weaning. Therefore, the objective of the current study is to compare the effects of feeding 2 milk replacers (20% CP and 20% fat [MR20] vs. 24% CP and 24% fat [MR24]) on calf growth, feed efficiency, and scouring frequency. Forty individually housed 2- to 5-d-old Angus × Holstein crossbred calves with a BW ranging from 34.0 to 47.6 kg were randomly assigned to 1 of 2 milk replacer (MR) treatments. Calves were fed 2.84 L of their respective MR twice per day (5.68 L total) from d 1 to 42 of the experiment and 2.84 L once per day from d 43 to 49. Feeding crossbred calves MR24 increased ADG and gain per feed by 23.6% and 9.80%, respectively, compared with calves fed MR20 from d 1 to 56. Feeding crossbred calves MR24 tended to increase calf starter intake and total feed intake compared with calves receiving MR20. Results suggest feeding a MR of higher nutritive value is a simple and effective strategy to increase weight gain and feed efficiency of beef × dairy calves.

## Highlights

• 24% CP and 24% fat milk replacer improved ADG in beef × dairy calves.
• 24% CP and 24% fat milk replacer increased gain per feed and energy and protein intake.
• 24% CP and 24% fat milk replacer increased starter intake during late preweaning.
• Fecal scores and scouring incidence were not affected by milk replacer composition.

## Graphical Abstract

**Figure:**
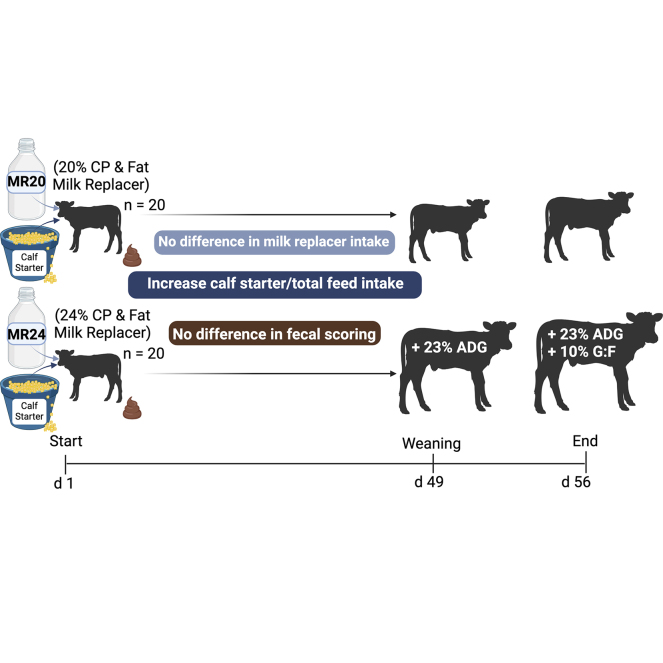
**Summary:** Best feeding practices have largely yet to be established for the increasing proportion of beef × dairy calves produced on dairy farms. Therefore, the current study sought to compare the effects of feeding milk replacers (MR) containing 20% (MR20) and 24% (MR24) crude protein (CP) and fat to beef × dairy calves. Forty calves received 1 of 2 MR treatments and calf starter (CS) starting from day 2 to 5 of age (day 1 of the experiment) until weaning 49 days later, at which point calves were offered solely CS until day 56. This study observed that the average daily gain (ADG) of crossbred calves receiving MR24 increased 23.4% and 23.6% during the preweaning and total experiment, respectively. Additionally, feeding MR24 to crossbred calves increased feed efficiency (gain/feed, kg/kg) by 9.80% during the entire 56-day period compared with MR20. Milk replacer treatment had no impact on fecal scores of beef × dairy crossbred calves during the preweaning period or entire experiment.

Advances in the last 30 years, such as improved genetics for fertility and estrous detection techniques, have drastically increased producers' ability to successfully produce replacement heifers for their herds (Lucy, 2023). Breeding females with lower genetic merit to beef sires allows better management of heifer inventories and creates higher value beef animals than purebred dairy steers. Lauber et al. (2023) reported that the proportional use of beef semen for breeding Holstein damns increased from 18.2% in 2019 to 26.1% to 2022. Beef × dairy calves are reared similarly to dairy bull calves destined for beef markets, but are known to have enhanced feedlot finishing performance, efficiency, and immune function compared with purebred dairy steers (Berry, 2021; Pereira et al., 2021). Given their greater genetic merit for growth, beef × dairy calves are typically sold for a premium relative to conventional dairy calves entering backgrounding operations (Wilson et al., 2020). Additionally, beef × dairy calves can be sold for quality premiums typically not associated with purebred dairy calves sold for beef (Ettema et al., 2017). Profitability of dairy operations breeding dairy dams to beef semen is nearly always greater than conventional or sexed dairy semen across different geographic regions and production system, which is reflected in the greater demand for beef × dairy calves at market (Basiel and Felix, 2022). For example, Pahmeyer and Britz (2020) reported that use of beef semen in dairy breeding programs can increase average profitability per cow by approximately €80 (US$87) per year for German dairy operations. Increased profitability from sales of beef × dairy cattle for beef production creates opportunities for dairy operations to diversify revenue and increase income.

Peterson et al. (1989) reported that larger beef cattle entering the feedlot had greater ADG than smaller cattle entering the feedlot. Preconditioning before entering the feedlot is an important factor influencing weight at the time of entering the feedlot, suggesting that this time period is crucial for subsequent success in the feedlot. A potential method for producing larger calves entering the feedlot could be to feed more nutrient-dense MR. Hill et al. (2008) observed a 53.8% increase in ADG when feeding Holstein heifer calves a milk replacer (**MR**) with greater protein and fat (27.1% and 27.6%, respectively) contents compared with a standard commercially available MR (19.8% and 21.1%, respectively). Although these results seem transferable to crossbred calves, data on how crossbred calves respond to a MR of greater protein and fat concentrations with regard to growth and overall health are limited. Understanding how to best feed preweaning crossbred calves is important for coordinated production systems seeking high quality, efficient cattle. Therefore, the objective of this study is to compare 2 MR containing 20% and 24% CP and fat on performance of beef × dairy crossbred calves for 7 wk preweaning and 1 wk postweaning. We hypothesize that crossbred calves fed MR containing 24% CP and fat will have greater ADG and reduced scouring compared with calves fed MR containing 20% CP and fat.

To test this hypothesis, a study was conducted at the University of Minnesota Southern Research and Outreach Center (**SROC**; Waseca, MN) Calf and Heifer Research Facility. Forty Angus × Holstein crossbred bull calves were continuously sourced from a local dairy farm (Goodhue, MN) between July and September and brought to SROC at 2 to 5 d of age. Enrollment criteria required calves to have received 3 feedings of colostrum (3.74 L/feeding) within 48 h of age, be free from apparent health issues, and have a serum total protein concentration greater than 5 g/dL and an initial BW between 34.0 and 47.6 kg upon arrival to SROC. Calves were housed individually in outdoor high-density polyethylene vented hutches (212 cm × 114 cm × 124 cm; Calf-Tel, Hampel Corp., Germantown, WI) with attached outdoor calf panel pens (total dimension of ∼3 m^2^), bedded with shavings refilled daily during the entire 56-d experiment. Upon arrival at the facility, calves were vaccinated intranasally for infectious bovine rhinotracheitis, parainfluenza 3, and bovine respiratory syncytial virus (Inforce-3, Zoetis Inc., Parsippany-Troy Hills, NJ). Feeding, BW measurements, and fecal scores were conducted by previously trained research staff at SROC. Housing, welfare conditions, and treatments were approved by the University of Minnesota Institutional Animal Care and Use Committee.

The experiment employed a completely randomized design with calves randomly assigned to 1 of 2 treatments: MR with 20% CP and fat (**MR20**) and MR with 24% CP and fat (**MR24**). Calf was the experimental unit (n = 20/treatment). Sample size was determined based on at least an 80% power of observing a *P* < 0.05 difference in our primary outcome measure ADG, based on an SD of 0.02 kg and an expected difference of 0.1 kg observed in previous experiments (Chester-Jones et al., 2016; Jaeger et al., 2020). Calves were randomly assigned to calf hutches to minimize confounding effects of location. Both MR prepared by Actus Nutrition (Eden Prairie, MN) had fat composed of tallow, lard, and coconut fat, and protein composed of whey protein concentrate, hydrolyzed wheat gluten, and bovine plasma (Table 1). From d 1 to 42 of the experiment, calves were fed 5.68 L of MR (12.5% wt/wt) per day in 2 feedings at 0500 and 1500 h. From d 43 to 49 of the experiment, 2.84 L of MR (12.5% wt/wt) was fed once per day at 0500 h, and calves were weaned on d 49. Starting on d 1 of the experiment, all calves were fed an 18% CP commercial calf starter (**CS**; Hubbard Feeds, Mankato, MN) containing 10.0 mg/kg diflubenzuron (ClariFly, Central Life Sciences, Schaumburg, IL), 50.0 mg/kg decoquinate (Deccox, Zoetis, Parsippany-Troy, NJ), and a standard vitamin and mineral mix ad libitum. Fresh water was provided to all animals ad libitum*.*

**Table 1:** Nutrient composition of milk replacer and calf starter for experiment determining the impacts of feeding milk replacer containing either 20% or 24% protein and fat on growth, intake, and scouring incidence of beef × dairy crossbred calves for 7 wk preweaning and 1 wk postweaning

Item	Milk replacer^1^		Calf starter
	MR20	MR24	
DM, %	96.6	96.6	90.8
CP, % of diet DM	20.1	23.3	22.4
ADF, % of diet DM	—	—	8.66
aNDF,^2^ % of diet DM	0.36	0.01	12.3
Starch, % of diet DM	—	—	35.2
Fat, % of diet DM	20.0	23.2	4.04
Ash, % of diet DM	6.62	6.26	7.57
Ca, % of diet DM	1.04	1.10	1.46
P, % of diet DM	0.73	0.78	0.69
Mg, % of diet DM	0.12	0.12	0.33
K, % of diet DM	1.53	1.50	1.41
S, % of diet DM	0.40	0.41	0.42
NEG, Mcal/kg	1.97	2.07	1.41

1 MR20 = milk replacer formulated to contain 20% CP and 20% fat; MR24 = milk replacer formulated to contain 24% CP and 24% fat.

2 Amylase- and sodium sulfate-treated NDF.

Upon arrival, blood samples were taken via jugular venipuncture into 10-mL serum separation tubes (BD Vacutainer, Rutherford, NJ), allowed to clot and were centrifuged at 3,000 × *g* for 20 min at ambient temperature. Serum protein concentration was immediately analyzed by a hand-held refractometer (Spartan Refractometer Model A 300 CL, Spartan, Tokyo, Japan). Serum protein at arrival was 6.30 ± 0.22 g/dL (µ ± SEM) for MR20 and 5.85 ± 0.20 g/dL for MR24 (*P* = 0.13). One 50-g sample of MR and CS were taken directly from each 22.7-kg bag fed and composited for analysis of chemical composition at a commercial laboratory (Dairyland Laboratories Inc., Arcadia, WI) according to AOAC International (2000) wet chemistry methods for DM (935.29); CP (976.06); ADF (973.18), amylase- and sodium sulfate-treated NDF (2002.04), starch (920.40), ether extract (989.05 for MR and 920.39 for CS); ash (942.05); and Ca, P, K, and Mg (985.01).

Each day, MR and CS were provided and refusals were weighed and subtracted to calculate feed ingredient and total intakes. Daily NEG and CP intakes were calculated by multiplying concentrations of these nutrients in each MR treatment and CS by their respective daily DMI for each calf, then summing to obtain total daily intake of each nutrient. Calves were weighed on d 1, 14, 28, 42, 49, and 56 of the experiment using a digital scale (Cardinal Model 205, Rice Lake Weighing Systems, Rice Lake, WI), and ADG was calculated. Gain per feed (**G:F**) was calculated by dividing ADG by total daily DMI (MR + CS). Gain per Mcal of NEG and per kilogram of CP intake were also determined. Hip height (**HH**) was determined on d 1, 49, and 56 of the experiment using a Nasco Dairy and Beef Measuring Stick (Ft. Atkinson, WI). Fecal consistency was visually evaluated during morning feeding using a 1 to 4 a scale (1 = normal; 2 = loose, pudding; 3 = very loose, no watery separation; and 4 = very watery; Larson et al., 1977). Daily fecal scores were averaged into 14-d periods that represented the same time frames as BW. Calves that were determined to be scouring (fecal score of ≥3) were treated with sulfamethazine without electrolytes.

A generalized linear model tested the effects of treatment, time interval, and the interaction of treatment and time interval on BW, HH, ADG, MR intake, CS intake, NEG intake, CP intake G:F, gain per NEG, gain per CP, and average fecal score using the *lm*() function of the *stats* package in R version 4.4.1 (https://www.r-project.org/). Age at enrollment and serum protein concentration at enrollment were used as covariates and removed from statistical models if their effects were not significant (*P* > 0.10). Age at enrollment was used as a covariate for BW, CS intake, NEG intake, CP intake, and HH. Serum protein concentration at enrollment was used as a covariate for fecal score, BW, HH, CS intake, NEG intake, CP intake. Least squares means and preplanned pairwise comparisons between treatments at each time point were determined using the *emmeans*() package of R. Total ADG, HH gain, MR intake, CS intake, G:F, average fecal score, and scouring frequency (days with a fecal score ≥3) over the preweaning period (d 1 to 49 of the experiment) and experiment (d 1 to 56) were analyzed as a linear model using the *lm()* package of R with the fixed effect of treatment. Significance was declared at *P* ≤ 0.05, and trends were declared at 0.05 < *P* ≤ 0.10. Heteroscedasticity was determined by generating a histogram and normal quantile-quantile plot of residuals.

Upon arrival (d 1 of the experiment), calf BW averaged 41.4 ± 1.71 kg (LSM ± SEM) for MR20 and 43.2 ± 1.71 kg for MR24 (*P* = 0.45; Figure 1A). Body weight was affected by treatment (*P* < 0.001) and day (*P* < 0.001) but not the interaction of treatment and day (*P* = 0.25), with BW being 10.7%, 12.9%, 10.3%, and 12.0% greater in MR24 at d 28, 42, 49, and 56 of the experiment, respectively. Average daily gain was also affected by treatment (*P* < 0.001) and time (*P* < 0.001), but not the interaction of treatment and time (*P* = 0.65; Figure 1C). Feeding beef × dairy calves MR24 increased ADG by 46.3% from d 15 to 28 (*P* = 0.03), tended to increase ADG by 21.0% from d 29 to 42 (*P* = 0.07), and increased ADG by 21.3% from d 50 to 56. Average daily gain was increased in MR24 during the preweaning period and experiment by 23.4% and 23.6%, respectively (*P* < 0.05).

**Figure 1:**
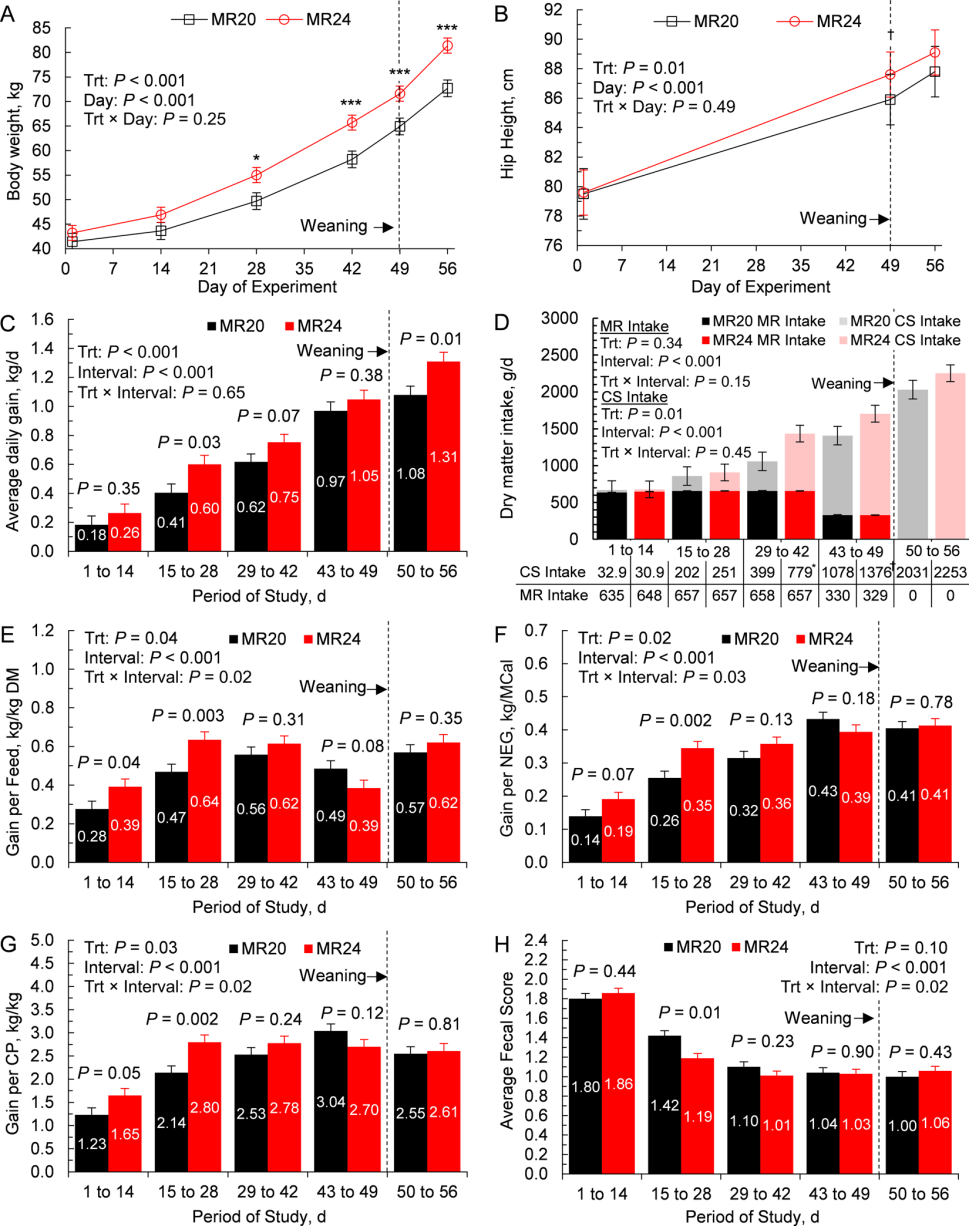
Effects of feeding beef × dairy crossbred calves MR containing either 20% or 24% protein and fat on (A) biweekly BW; (B) hip height on d 1, 49, and 56; (C) biweekly ADG; (D) biweekly DMI; (E) biweekly gain per feed; (F) biweekly gain per NEG; (G) biweekly gain per CP; and (H) and average biweekly fecal score for 7 wk preweaning and 1 wk postweaning. Treatments included a 20% fat and 20% protein milk replacer (MR20) and a 24% fat and 24% protein milk replacer (MR24). Calves in both treatments were fed 5.67 L of MR (12.5% wt/wt) per day from d 1 to 43, 2.83 L of MR (12.5% wt/wt) per day from d 43 to 49, and were weaned on d 49. Data are presented as LSM with bars indicating SEM. For panels A and B, days denoted with *** were considered different at (*P* ≤ 0.01), days denoted with * were considered different (0.01 < *P* ≤ 0.05), and days denoted with † were considered to exhibit a tendency for difference (0.05 < *P* < 0.10). For panel D, treatments that differed (*P* ≤ 0.05) or tended to differ (0.05 < *P* < 0.10) in MR or CS intake within each time interval were denoted with * or †, respectively, next to the larger of the 2 means.

Hip height was similar (*P* = 0.92) for MR treatments on d 1 of the experiment, with MR20 starting on the experiment with an average HH of 79.5 ± 0.69 cm and MR24 starting with an average HH of 79.6 ± 0.69 cm (Figure 1B). Treatment (*P* = 0.01) and day (*P* < 0.001) affected HH, but not the interaction (*P* = 0.49), with d-49 HH tending to be greater for MR24 than MR20 (*P* = 0.08). The MR24 treatment increased and tended to increase HH gain for the preweaning and entire experiment by 24.9% (*P* = 0.02) and 14.5% (*P* = 0.09), respectively (Table 2).

**Table 2:** Effects of feeding milk replacer containing either 20% or 24% protein and fat on ADG, hip height gain, feed intake, feed efficiency, and scouring incidence of beef × dairy crossbred calves during the 49-d preweaning period and entire experiment

Item	Treatment^1^		SEM	*P*-value^2^
	MR20	MR24		
ADG, kg/d				
Preweaning^3^	0.47	0.58	0.037	0.05
Experiment^4^	0.55	0.68	0.036	0.01
Hip height gain, cm/d				
Preweaning	6.38	7.97	0.45	0.02
Experiment	8.32	9.53	0.49	0.09
Milk replacer intake, g/d				
Preweaning	604.5	607.5	2.47	0.39
Calf starter intake, g/d				
Preweaning	323.2	504.2	68.3	0.07
Experiment	528.7	723.4	73.0	0.07
Total feed intake, g/d				
Preweaning	927.6	1,112	69.0	0.07
Experiment	1,058	1,255	73.6	0.07
NEG intake, Mcal/d				
Preweaning	1.66	1.87	0.066	0.03
Experiment	1.82	2.03	0.078	0.04
CP Intake, g/d				
Preweaning	207.1	239.9	10.4	0.02
Experiment	237.4	286.9	15.0	0.03
Gain per feed, kg/kg DM				
Preweaning	0.49	0.54	0.024	0.21
Experiment	0.51	0.56	0.017	0.05
Gain per NEG, kg/Mcal				
Preweaning	0.26	0.31	0.011	0.01
Experiment	0.28	0.31	0.010	0.02
Gain per CP, kg/kg				
Preweaning	2.10	2.37	0.080	0.02
Experiment	2.16	2.37	0.070	0.04
Average fecal score				
Preweaning	1.37	1.30	0.037	0.21
Experiment	1.32	1.27	0.030	0.25
Scouring frequency, d				
Preweaning	1.21	0.80	0.26	0.28
Experiment	1.21	0.80	0.26	0.28

1 MR20 = milk replacer formulated to contain 20% CP and 20% fat; MR24 = milk replacer formulated to contain 24% CP and 24% fat.

2 *P*-value of main effects of milk replacer fat and protein concentration.

3 The preweaning period encompassed d 1 to 49 after enrollment on trial.

4 The entire experiment included the 49-d preweaning period and 7 d postweaning.

Milk replacer intake was not affected by treatment (*P* = 0.34; Figure 1D) and averaged 604.5 ± 2.47 g/d for MR20 and 607.5 ± 2.47 g/d over the preweaning period (Table 2). However, CS intake was affected by treatment (*P* = 0.01), with MR24 increasing intake by 95% from wk 29 to 42 (*P* = 0.03), and tending to increase intake from wk 43 to 49 (*P* = 0.08). Total daily CS intake across the preweaning period and experiment tended to be increased by 56% (*P* = 0.07) and 36.8% (*P* = 0.07), respectively, in calves receiving MR24. Total DMI (MR + CS) was affected by treatment (*P* = 0.01), with a tendency for greater intake from d 29 to 42 (1,060 vs. 1,440 ± 157 g/d; *P* = 0.09) and a 32% increase from d 43 to 49 (2,079 vs. 2,761 ± 157 g/d; *P* = 0.003) in MR24. Total daily DMI during the preweaning period and the entire experiment tended to increase by 14.0% (*P* = 0.07) and 12.9% (*P* = 0.07), respectively, for beef × dairy calves receiving MR24.

Intake of NEG was affected by treatment (*P* = 0.001), time interval (*P* < 0.001), and their interaction (*P* < 0.001; data not shown), with MR24 having greater NEG intake from d 43 to 49 (2.18 vs. 2.62 ± 0.13 Mcal/d; *P* = 0.02), and a tending to have greater intake from d 50 to 56 (2.88 vs. 3.17 ± 0.13 Mcal/d; *P* = 0.10). Greater NEG intakes of 12.7% and 11.5% occurred in MR24 across the preweaning period (*P* = 0.03) and entire experiment (*P* = 0.04), respectively (Table 2). Similarly, CP intake was affected by treatment (*P* = 0.001), time interval (*P* < 0.001), and their interaction (*P* < 0.001; data not shown). Intake of CP was greater for MR24 during d 43 to 49 (315 vs. 384 ± 20.2 g/d; *P* = 0.02), and tended to be greater from d 50 to 56 (456 vs. 503 ± 20.2 g/d; *P* = 0.10). Across the preweaning period, CP intake was 15.8% greater in MR24 (*P* = 0.02; Table 2). Moreover, CP intake was 20.9% greater in MR24 across the full experiment (*P* = 0.03).

Feed efficiency, measured as gain per feed (kg/kg DM), was affected by treatment (*P* = 0.04), time interval (*P* < 0.001), and the interaction of treatment by time interval (*P* = 0.02; Figure 1E). Gain per feed was 39.3% greater in MR24 from d 1 to 14 (*P* = 0.04) and 36.2% greater in MR from d 15 to 28 (*P* = 0.004). From d 43 to 49, G:F tended to be greater in MR20 than MR24 (*P* = 0.08), due to the greater feed intake during this interval. Gain per feed was not different across the preweaning period (*P* = 0.21) but was greater in MR across the entirety of the experiment (*P* = 0.05; Table 2).

Energy efficiency, measured as gain per NEG intake (kg/Mcal) was affected by treatment (*P* = 0.02), time interval (*P* < 0.001), and the interaction of treatment and time interval (*P* = 0.03; Figure 1F), with a tendency for greater efficiency in MR24 from d 1 to 14 (*P* = 0.07), and a greater efficiency in MR24 from d 15 to 28 (*P* = 0.002). Gain per NEG was also greater in MR24 across the preweaning (*P* = 0.02) and experimental (*P* = 0.04) periods (Table 2). Nitrogen efficiency, determined as gain per CP intake (kg/kg) was also affected by treatment (*P* = 0.03), time (*P* < 0.001), and their interaction (*P* = 0.02), with greater gain per CP in MR24 from d 1 to 14 (*P* = 0.05) and d 15 to 28 (*P* = 0.002; Figure 1G). Across the preweaning period, and experimental period gain per CP was 12.9% and 9.27% greater in MR, respectively (*P* < 0.04).

Fecal score tended to be affected by treatment (*P* = 0.10), and was affected by time interval (*P* < 0.001), and their interaction (*P* = 0.02; Figure 1H). The MR24 treatment decreased average fecal score by 16.2% compared with MR 20 (*P* = 0.01). Average fecal score across the preweaning and experimental period did not differ by treatment (*P* ≥ 0.21; Table 2). Similarly, frequency of scouring (days with a fecal score ≥3) did not differ between treatments within the preweaning period (*P* = 0.28). No scouring occurred within the postweaning period.

The primary objective of the current study was to evaluate the impacts of feeding 2 MR of different protein and fat contents on beef × dairy calf growth and health. Although the study is relatively straightforward in design, it addresses a financially important question applicable to a growing proportion of dairy operations. As beef × dairy breeding programs become more prominent, tailored nutritional programs to optimize growth and health of these crossbred calves would be advantageous for producers to implement to maximize profits from calf sales.

As hypothesized, calves fed MR24 had greater final BW gain and ADG compared with those fed MR20. Although data are currently lacking on optimal feeding practices specific to beef × dairy calves, studies evaluating performance of purebred Angus or Holstein calves can serve as references. Our BW and ADG results agree with Hill et al. (2008), who observed greater final BW and ADG for purebred Holstein calves fed MR of a greater protein and fat content. Growth in calves is largely driven by quantity of nutrients consumed (Chapman et al., 2016). Although MR24 calves gained more weight over the course of the entire experiment (d 1 to 56) compared with MR20 calves, ADG responses between the 2 treatments were less pronounced during the week of weaning (d 43 to 49). This response in ADG is likely due to a decrease in MR intake from d 43 to 49 (weaning), which diluted the effect of the greater protein and fat content of MR24 and caused nutrient intake across treatments to be similar. These results agree with Suarez-Mena et al. (2021), who increased nutrient intake of purebred Holstein calves by feeding MR at 2 levels of intake and reported calves with higher nutrient intake had greater ADG before weaning. Increases in ADG, especially during the preweaning period, could also be attributed to greater frame growth, as HH gain of calves fed MR24 was substantially greater than calves fed MR20. Hill et al. (2009a) also reported that increasing protein intake of young calves via MR increased HH, suggesting greater frame and organ growth as a result of greater protein intake. Although increased fat intake has been shown to restrict CS intake of calves (Hill et al., 2009b), this did not occur in our study, potentially due to greater development early in life.

Feed efficiency on a total DMI basis, a NEG basis, and a CP basis was greater for MR24 calves compared with MR20 calves over the course of the entire experiment (d 1 to 56) which is in agreement with Quigley et al. (2006). However, the increase in feed efficiency associated with feeding MR24 declined over the course of the experiment, especially from d 29 to 42, when MR20 had statistically greater G:F, and d 43 to 49 which had numerically greater G:F, gain per NEG and gain per CP. The substantial increase in feed efficiency during the first 28 d of the experiment were strong enough to drive an increase in feed efficiency for the entirety of the experiment. These findings suggest feeding beef × dairy crossbred calves a MR of a higher nutritional value allows for greater ADG during the preweaning period; however, the efficiency of this gain might be diminished as the calf approaches the age of weaning.

Fecal scoring is typically used to evaluate prevalence of diarrhea in calves, which is associated with reduced growth rates and lower subsequent production later in the animal's life (Renaud et al., 2020). Calves can suffer from diarrhea if energy intake is insufficient (Owen et al., 1958) or if protein and fat intake from MR exceed the calf's ability to digest and absorb the nutrients (Kertz et al., 2017). A fecal score of 3 or greater indicates diarrhea, which rarely occurred for either treatment in this experiment. Animals on both treatments were generally healthy and had enough energy to maintain immune function throughout the preweaning period. In support of the current study, Hill et al. (2008) also did not report any major differences in fecal scores when feeding milk replacers of greater protein and fat concentrations.

Substantial improvements in ADG and feeding efficiency throughout the entirety of the 56-d experiment can be attributed to the greater intake of protein and fat for crossbred calves fed MR24 as intakes of MR and CS were similar. This promising feeding strategy could be easily implemented on dairy herds to increase subsequent performance of crossbred calves in finishing operations as these calves achieved greater and more efficient gain compared with MR20 calves, 2 key indicators of future success in feedlots.
